# Supplementary material for: Detecting and describing stability and change in COVID-19 vaccine receptibility in the United Kingdom and Ireland
Source: PLoS One. 2021 Nov 3;16(11):e0258871. doi: 10.1371/journal.pone.0258871 (PMC8565732; doi:10.1371/journal.pone.0258871)
Supplement: Attachment — Submitted filename: 2. Response to Reviewers.docx [file pone.0258871.s001.docx]

**Response to reviewer comments**

Reviewer #1: Because of the hotness of the focal topic; about the acceptance of a COVID-19 vaccination, the report work might be informative and meaningful to the world-wide audience. The science of their survey in terms of ethics, procedure, statistics, and other analysis, looks healthy. Therefor, I evaluate the work with a positive impression.

The suggestions are given as below to improve the final form of contribution.

**Reply**: Thank you.

#1.
The main result about the acceptance of each of those regions (two nations) and its time-series in the real wake of COVID-19 is quite interesting. If it is possible, the audience including me would love to hear why the acceptance level amid those two countries being different, which can be referring to unvisitable, potential and social reasons in view of comparison between those two close neighboring countries (closes each other).

**Reply**: We understand the request to discuss differences in vaccine acceptance rates between Ireland and the UK but because this was not one of our study objectives, we did not perform any tests of differences across countries. Therefore, we would have to resort to speculation as to why these differences exist. We believe that this would serve to distract for the discussion of the key study results. Moreover, the reason we did not formulate specific research objectives relating to cross-country comparisons in the first place is that we wished to examine and identify the contextually specific risk factors associated with vaccine hesitance and resistance in each country so that public health agencies in these countries could tailor interventions and messaging that is appropriate for their respective populations. This issue is discussed on page 22 line 525.

#2.
The acceptance of vaccination, which might be quantified by each of responders with reference to various factors, as the authors discussing, such as benefic (vis-à-vis thread of infection), risk of side-effect, and other things, should be more profoundly discussed. One important aspect is whether such acceptance of vaccination can be predicted by a model framework in the help from applied mathematical framework such as Evolutionary Game Theory and others. Citing several precursors; for example, ‘Hypothetical assessment of efficiency, willingness-to-accept and willingness-to-pay for dengue vaccine and treatment: a contingent valuation survey in Bangladesh, Journal Human Vaccines & Immunotherapeutics, 2020’, the authors should supplement further discussion.

**Reply**: Thank you for bringing this excellent paper to our attention. We read it with great interest. Although an excellent piece of work, we do not think it is especially relevant to this paper because the COVID-19 vaccines are being offered to the public (at the very least in the countries assessed in this study) free of charge.

Reviewer #2: This paper is well written, the topic is interesting, and the results seem correct; the work is acceptable. I make some recommendations for minor revisions. Besides these issues, the work appears methodologically sound and is well written.

**Reply**: Thank you.

##1
Your abstract looks very technical level, and it isn't easy to understand the key point of your findings. I want to ask you to rewrite the abstract. For instance, the abstract describes the framework but leaves it to the reader to dig into the Results to find out what is novel about the predictions. I think the work would be better served if the abstract highlighted some of the most important findings emerging from this model. Or, what novel/surprising results emerged from this work?

**Reply**: Thank you for the opportunity to revise the abstract. We have amended the abstract considerably, taking advantage of the 300-word limit for the abstract to provide more concrete details on the background of the study and the key findings. We believe the abstract is clearer now.

##2
If possible, please add one figure that can represent overall picture of study settings and findings for general readers.

**Reply**: We do not believe it possible to summarise all of the methodological and statistical information in a single figure. Indeed, this information is distributed across all of the tables and figures in our submission.

##3
The current form of figure is terrible, less-explanation, unclear legend, unsmooth colored and the range of parameters. Would you please make clear the figures?
Figure captions: please re-write the figure caption in detail that can easily be persuasive to a reader.

**Reply**: We respectfully disagree. We believe the figures are clear, easy-to-understand, and the legends provide an accurate representation of the content of the figures. We consulted numerous papers on this topic, and papers using similar methodologies, and found that our figures are very much in line with standard practice in the field. We do not wish to be difficult, and would happily revise this if the reviewer has specific suggestions and examples the he/she could provide.

##4
In the introduction, authors should cite the following literatures,

Hypothetical assessment of efficiency, willingness-to-accept and willingness-to-pay for dengue vaccine and treatment: a contingent valuation survey in Bangladesh, Human vaccine and Immunotherapeutics, DOI: 10.1080/21645515.2020.1796424 (2020).

Evolutionary game theory modelling to represent the behavioural dynamics of economic shutdowns and shield immunity in the COVID-19 pandemic. R. Soc. Open Sci. 7: 201095. <http://dx.doi.org/10.1098/rsos.201095> (2020).

A cyclic epidemic vaccination model: Embedding the attitude of individuals toward vaccination into SVIS dynamics through social interactions, Physica A, 581, 126230 (2021).

**Reply**: We have reviewed all three papers and do not believe they are relevant to our study. We also note that all three papers are authored by the same two people.
